# HIV-1 and HIV-1-Tat Induce Mitochondrial DNA Damage in Human Neurons

**DOI:** 10.16966/2380-5536.176

**Published:** 2020-08-31

**Authors:** Nune Darbinian, Armine Darbinyan, Nana Merabova, Michael E Selzer, Shohreh Amini

**Affiliations:** 1 Center for Neural Repair and Rehabilitation, Lewis Katz School of Medicine, Temple University, Philadelphia, USA; 2 Department of Pathology, Yale University School of Medicine, New Haven, USA; 3 Department of Biology, College of Science and Technology, Temple University, Philadelphia, USA

**Keywords:** mtDNA, HIV-1, Tat, Human neurons, mtDNA damage

## Abstract

**Introduction:**

Mitochondrial dysregulation is a key event in HIV-1 infection. Recent studies have suggested that age-related neurodegenerative disorders are associated with increased mitochondrial DNA (mtDNA) damage. As accelerated ageing was found in HIV-1 patients, we hypothesized that HIV-1 infection or HIV-1 proteins can lead to mtDNA damage. Unrepaired mtDNA impairs mitochondrial function, which can lead to oxidative stress and cell death. Investigations of mechanisms of mtDNA damage are limited by the lack of available human models.

**Methods:**

We compared mtDNA or nDNA (nuclear DNA) damage in human cortical neurons and PBMC cells. Primary neuronal cultures were incubated with conditioned media from HIV-1 infected PBMC, or HIV-1 viral proteins Tat or Vpr. Total genomic DNA (nuclear and mtDNA) was isolated using the QIAamp Kit. Nuclear and mtDNA were amplified using the long q-PCR/Gene Amp XL Kit. Real-Time RT-PCR using mitochondrial energy metabolism array was performed to assess mitochondrial energy metabolism markers. Superoxide dismutase (SOD) activity in neuronal cells was measured by the OxiSelect SOD Activity Assay. Reactive oxygen species (ROS) were determined by the confocal microscopy. ATP levels were analyzed using ATP determination biochemical assay. Mitochondrial, cytoplasmic and nuclear proteins were studied by quantitative western-blot assay.

**Results:**

We show that both treatment of neuronal cells with HIV-1 conditioned media, or infection of PBMC with HIV-1 increase mtDNA damage in cells. mtDNA damage was also seen in neuronal cells, incubated with HIV-1 proteins, Tat and Vpr. Next, we confirmed that mtDNA damage was also increased in neuronal cells transfected by Tat expressing plasmids. We showed that mtDNA was not damaged in neuronal cells following treatment with heat inactivated HIV-1 or Tat protein. Further, we demonstrated that HIV-1 or Tat caused more mtDNA damage compared to nuclear DNA damage in neuronal cells. Finally, we showed that Tat dysregulates RNA expression of several genes regulating mitochondrial energy metabolism, suggesting involvement of Tat in mitochondrial bioenergetics in human neurons. Finally, our hypothesis was confirmed by qWestern analysis of mitochondrial and apoptotic proteins demonstrating the accumulation of apoptotic Bax and Bad proteins in mitochondrial fraction of Tat-treated neuronal cells, suggesting toxic effects of Tat on mitochondrial survival.

**Conclusion:**

We showed an increase of mtDNA damage in primary neurons, treated with HIV-1 proteins and in PBMC, infected with HIV-1. Increased mtDNA damage can lead to neurodegeneration, and cause neuronal apoptosis. Our system presents a suitable model to study mtDNA changes during HIV-1 infection.

## Introduction

Mitochondria represent one of sources of increased levels of reactive oxygen species (ROS) in age-related neurodegenerative disorders [1]. ROS increase is correlated with mitochondrial DNA (mtDNA) damage [2]. Mitochondrial oxidative phosphorylation in PBMC is inhibited in chronic infection by HIV-1, and is correlated with immune dysregulation [3]. It was shown that HIV-1 Tat inhibits survival of neuronal cells *via* mitochondrial dysfunction, and alterations in mitochondrial oxidative phosphorylation can be an essential feature of HIV-1 pathogenesis [4]. HIV-1 infection has various effects on mitochondria [5], and was shown to decrease mtDNA in PBMCs [6–9]. HIV-1 Tat increases DNA damage in human peripheral blood B-lymphocytes by the production of mitochondrial ROS [10]. HIV-1 Tat and gp120 proteins can increase mitochondrial fragmentation in human neurons [11]. Furthermore, HIV-1 Tat can cause mitochondrial dysfunction in activated microglia [12]. It was also established that another HIV-1 product, auxiliary regulatory protein, Vpr, could induce activation of the ATR-initiated DNA damage-signaling pathway leading to phosphorylation of Chk1, and the formation of gamma-H2AX and 53BP1 nuclear foci [13–16]. Further, Vpr was shown to execute its role in DNA damage response *via* interaction with the damaged DNA-binding protein (DDB1) and Vpr-binding protein (VprBP), a binding partner for DDB1 [17]. In addition, major components of highly active antiretroviral therapy (HAART), nucleoside analogue reverse-transcriptase inhibitors (NRTIs), inhibit *in vitro* DNA polymerase-γ, which controls mtDNA replication [18]. The mitochondrial genome is 16.6 kb in length, and encodes 37 genes: two rRNAs, 22 tRNAs, and 13 protein subunits. All these molecules are involved in the regulation of oxidative phosphorylation. Mitochondrial genes are maternally inherited, and the majority (99%) of mitochondrial proteins are regulated by nuclear DNA (nDNA), translated in the cytoplasm and then transported to the mitochondrial compartment [19]. Mitochondria are being replaced within 10-25 days, and old mitochondria are replaced by autophagy (mitophagy). HIV-1 affects mitochondrial fission/fusion in the neurons during HIV-related neurocognitive disorders [20]. Each cell contains up to thousands of mtDNA molecules. mtDNA is organized in nucleoid structures, each nucleoid contains 2-10 mtDNA molecules and several mitochondrial proteins. mtDNA in brain has a long half-life of 25 days. mtDNA is replicated and transcribed within the mitochondria, independently from the nuclear DNA replication by the nuclear encoded mtRNA polymerase, mtDNA Polymerase-γ. mtDNA replication takes about one hour, and it involves regulation of mtDNA copy number. Interestingly, nDNA is less susceptible to oxidative damage than mtDNA. Nucleoside Reverse Transcriptase Inhibitors (NRTIs), cause defective mtDNA replication by inhibiting Pol-γ mtDNA replication peaked after 72 h in cortical neurons. Each mitochondrion contains 4-10 DNA molecules, and each cell has 1,000-10,000 copies of 16.5 kb of circular DNA.

nDNA was shown to be less susceptibility to DNA-damaging agents, than mtDNA in age-related neuro degenerative disorders [21], and nDNA damage is less extensive than mtDNA damage in human cells after oxidative stress [2]. The DNA repair mechanism for DNA damage in mitochondria is the Base Excision Repair (BER) pathway. Accumulation of mtDNA damage and ROS-induced mtDNA lesions correlates with age-related decrease in BER, and in *8-oxoguanine DNA glycosylase-1* (*OGG1*), an enzyme that can repair 8-oxoG DNA lesion [1].

There is much less known about neuronal mtDNA during HIV-1 infection, than nuclear DNA. Several recent studies demonstrate the effects of Tat on mitochondrial bioenergetics, although there is no information regarding the direct effect of Tat and HIV-1 on the mitochondrial DNA damage in human neurons. Furthermore, the effect of Tat on mtDNA levels and mitochondrial function in PBMC was recently described [22]. Effects of Tat on mitochondrial bioenergetics was also demonstrated in cardiomyocytes [23]. Furthermore, the effects of Tat on mitochondrial genes and DNA were shown in Jurkat cells [22]. Other studies discuss the effects of Tat on DNA damage in human peripheral blood B-lymphocytes *via* mitochondrial ROS production [10]. Finally, it was recently shown that Tat induces mitochondrial membrane permeabilization in human liver and heart [24]. Thus far, no direct effects of Tat or HIV-1 infection on mtDNA damage were demonstrated in human neurons.

Our goal was to examine the effects of Tat on neuronal mitochondrial DNA, as one of potential events in neurodegeneration and neurological complications in NeuroHIV.

Further studies are necessary to investigate neuronal mtDNA damage during infection, to uncover the role of HIV and HIV-1 proteins, Tat or Vpr on mtDNA damage and mitochondrial bioenergetics. To answer this question, we used human primary neuronal culture model and human PBMC to study mtDNA damage during HIV-1 infection, or caused by HIV Tat and Vpr proteins.

## Materials and Methods

### Cell culture

Human primary cortical neurons were prepared in our laboratory by Dr. Darbinyan [4,25–28]. Human fetal brains resulting from elective abortion were obtained from Advanced Bioscience Resources (ABR), Inc., Alameda, CA 94501 USA, under a protocol approved by Temple University’s IRB. The protocol also complied with NIH guidelines at the time, although currently, NIH guidelines do not permit new collection of human fetal tissues. In brief, 16-weeks fetal brain (approx 13 g) was collected and treated with Tryple Express enzyme (Invitrogen, CA), DNase I (10 U/mL; Sigma, St. Eouis, MO) for 15 min at 7°C, then washed three times with Hibernate E medium. Neurobasal medium containing B27 supplement and 0.25 mM Glutamax was used for a tissue trituration with a glass Pasteur pipette, and cells were then plated on poly-D-lysine-coated 60-mm dishes (Sigma). After 16 hours, 1 μM of Cytosine arabinoside Ara-C (Sigma) was added to cells for 48 hours. Cells were cultured in Neurobasal medium containing several antibiotics, including 10 μg/mL gentamycin, 100 units/mE penicillin and 10 μg/mL streptomycin. Medium also contained one μg of antifungal fungizone (Life Technologies, Inc.). Cells were maintained at 37°C incubator.

### Treatment of neuronal cells

Neuronal cells were treated with rTat 101 (ImmunoDiagnostics, Inc,), (50 ng/mL, or 3.6 nM), or conditioned media (CM) from HIV-1 infected PBMC for total of 56 hours. Twenty ng of Rotenone R 8875 (Sigma) were used as a positive control for mtDNA damage.

### Plasmids

The pEYFP-C1 (BD Biosciences Clontech) and CFP-Tat plasmids were previously described by Darbinian-Sarkissian N, et al. [29].

### Transfection

Transfection of neuronal cells was performed using Lipofectamine 2000 (Invitrogen, CA) and 5 μg of plasmid DNA, according to the manufacturer’s instructions, in 105 cells plated on 60 mm plates. Each transfection was repeated a minimum of two times with different plasmid preparations, in triplicates for each sample.

### Infection of PBMC with HIV-1

PBMC was cultured from collected Buffy Coat according to the IRB protocol approved by Temple University. Infection of PBMC was performed using the JR-FL strain of HIV-1 as previously described [30]. Cells were grown in RPMI media. Fifty ng of p24-containing virus stock were added to 1 × 10^6^ cells. First, cells were incubated with the viral stock in a serum-free medium for 2 h at 37°C, and were then washed twice with PBS. In parallel, uninfected control cells were prepared four days post-HIV-1-infection. Cells were harvested and analyzed by p24 ELISA. Heat inactivation of the virus was performed at 56°C for 2 hours. Samples were constantly mixed while were heated. The virus was then incubated on ice, and was used within 3 hours.

### p24 ELISA

Supernatants were collected five days post-infection, and assayed by p24 ELISA for the presence of viral p24, using p24 ELISA kit (BioChain Institute, Inc., CA). The samples were assayed by spectrophotometer using a 450 nm filter, and the presence of HIV p24 was confirmed in samples.

### Analysis of mtDNA damage by qPCR

QPCR for nuclear (nDNA) and mitochondrial (mtDNA) DNA integrity was carried out with GeneAmp XL-PCR kit (Applied Biosystems) that allows performing long PCR for up to 20 kb DNA products.

### Estimation of DNA damage

Quantification of PCR products and calculation of lesion frequency were done by using Pico [31–34].

### DNA isolation and qPCR

Genomic DNA was purified using the QIAamp DNA isolation kit (Qiagen, Chatsworth, CA) to perform long PCR [35,36]. QPCRs were performed using the GeneAmp PCR System 2400 using the GeneAmp XL PCR kit. Fifteen ng of genomic DNA, 100 μg/mL nonacetylated BSA, 0.2 mM DNTPs, 0.2 μM primers, and 1 unit of r*Thermus thermophilus* DNA polymerase were combined to perform long qPCR. The PCR cycle started with a 75°C hot-start. An initial denaturation was for 1 min at 94°C, and then 25 cycles contained 15 sec at 94°C denaturation and 12 min at 68°C primer extension. A final 10 min extension at 72°C was performed at the completion of the cycle. Products of PCR were shown by gel electrophoresis in a 1% agarose gel for four hours at 80 V in TBE (90 mM Tris/64.6 mM boric acid/2.5 mM EDTA, pH 8.3).

### Primers

Primer nucleotide sequences for the 17.7-kbβ-globin gene (GenBank: J00179), βGlobinf 5’-TTGAGACGCATGAGACGTGCAG-3’, βGlobin-r 5’-GCACTGGCTTAGGAGTTGGACT-3’; βGlobin-f 5’-CGA GTA AGA GAC CAT TGT GGC AG; and for the 16.2-kb fragment of the mitochondrial genome (J01415), MtL-f 5’-TGAGGCCAAATATCATTCTGAGGGGC-3’; MtL-r 5’-TTTCATCATGCGGAGATGTTGGATGG-3’ (RH1066), and Mt Short reverse primer: 5’-GTAGCCTCCTCAGATTCATTGAAC-3’.

### Lambda DNA

1 ng per 100 mkl reaction of 20.8 kb DNA target from 2 × 10^7^ starting copies; 4 mkl/lane (like 0.3 mkg). Genomic DNA-1 μg per 100 μL reaction.

### DNA lesion frequencies

These can be calculated as the amplification of damaged DNA (AD) that is normalized to the amplification of non-damaged controls (AO), which is resulting in a relative amplification ratio. Assuming a random distribution of lesions and using the Poisson equation, the average lesion frequency per DNA strand was determined: *λ* = −ln *A_D_/A_O_*

### Lesions

Lesions (Break frequency, BF)=−ln of mtDNA band intensities (treated/control) were calculated using the Poisson equation as described: the average lesion frequency per strand can be calculated as *λ* = −ln *A_D_/A_C_*. 1 lesion/mtDNA molecule (16.5 kbp) is considered as 0.061 lesion for each 1 kbp fragment amplified for Relative Amplification.

### Mitochondrial copy number: Fluorescence images of each transcript

This was detected at a wavelength of 530 nm. Quantification of mitochondrial gene copies was performed by the ratio of each mitochondrial transcript mean concentration to the mean housekeeping ribosomal or globin gene concentration of each sample [33].

### RNA preparation

RNA was isolated from human neurons using the total RNA Isolation Kit (Ambion, TX, USA). One μg of RNA was used for cDNA synthesis using reverse transcriptase (Roche Molecular Biochemicals, Indianapolis, IN, USA). The amplified DNA was analyzed by gel electrophoresis using a 2.0 % agarose gel.

### RT-PCR

The Superscript III One-Step RT-PCR System with Platinum *Taq* (Invitrogen, Carlsbad, CA, USA) was used. One microgram of total RNA and primers specific to *ogg1* gene were used to amplify OGG1. Three-step cycling, and separate annealing and extension steps were performed: **1)** cDNA synthesis and pre-denaturation at 55°C for 30 minutes; **2)** 40 cycles of PCR amplification with 15-second denaturing at 94°C, 30-second annealing at 60°C and 1 minute extension at 68°C; and **3)** final extension at 68°C for 5 minutes. The amplified DNA was analyzed by DNA gel electrophoresis using a 2.0 % agarose gel.

### Real-time qRT-PCR

Total RNA was isolated using the RNeasy kit (Qiagen, Valencia, CA). The RT-PCR reaction was performed with one μg total RNA, using One-Step FAST RT-PCR SYBR Green PCR Master Mix (Qiagen). StepOnePlus Real-Time PCR system thermo cycler was used. (Applied Biosystems). PCR conditions were as follows; activation 95°C 5 min, PCR 45 cycles: 95°C 10 sec, 60°C 20 sec, 72°C 30 sec, melting curve (95-65°C), cool to 40°C 30 sec. [37]. For relative quantification, the expression level of genes was normalized to the housekeeping gene β-actin. Results were in presented as arbitrary units and shown as % of control.

### Preparation of protein extracts

Cells were washed with ice-cold TNN buffer (Sigma) and 1% protease inhibitors cocktail (Sigma). Cell debris was removed by centrifugation for 5 min at 4°C (14,000 RPM). Ten micrograms of proteins were heated at 95°C for 10 min and separated by 10% sodium dodecyl sulfate-polyacrylamide gel electrophoresis (SDS-PAGE).

### Mitochondrial extract preparation

Mitochondrial extracts were prepared from 50 × 10^6^ cells using mitochondrial extraction kit (Imgenex).

### Western-blot assay

Protein samples were resolved by SDS/PAGE and transferred to supported nitrocellulose membranes (Bio-Rad) for 2 h at 4°C in transfer buffer (25 mM Tris pH 7.4, 193 mM glycine, 20% methanol) as described [29]. After blocking membranes for 30 minutes at room temperature with 10% nonfat dry milk in PBS-T (1 × PBS/0.1% Tween-20), membranes were washed and incubated with a 1:1000 dilution of primary antibodies for 2 h in 5% nonfat dry milk. After three times washing, blots were incubated with secondary anti-mouse or anti-rabbit antibodies conjugated to horseradish peroxidase (1:10,000 dilutions). Proteins were visualized with the enhanced chemiluminescence detection system, ECL+ (Amersham Pharmacia) and exposed to X-ray film.

### Quantitative western-blot assay

Mitochondrial proteins (10 μg) were diluted with 6 × Laemmli SDS-sample reducing buffer, heated at 95°C for 10 min and separated by gradient SDS-PAGE gel electrophoresis (4-20%) in 1X Tris-Glycine-SDS buffer and transferred to nitrocellulose membrane (Bio-Rad) and blotting papers (Grade GB003) for 2 h at 4°C. The blots were washed three times and proteins were detected using specific primary antibodies and secondary IRDye® antibodies with the Odyssey® CLx Imaging System. For the LI-COR system, blots were incubated with IRDye® 800CW Goat Anti-Rabbit and IRDye® 680RD Goat Anti-Mouse Li-COR dyes and visualized with an Odyssey® CLx Imaging System (LI-COR, Inc., Lincoln, NE) using Odyssey software (LI-COR Biosciences, Lincoln, NE, USA).

### Antibodies

Antibodies to Lamin A, VDAC, Bax, Bad and Cytochrome C were obtained from Santa Cruz Biotechnologies (Santa Cruz, CA). Mouse monoclonal Grb2 was obtained from BD Biosciences (San Jose, CA), anti-α-tubulin clone B512 was obtained from Sigma-Aldrich (Sigma-Aldrich Co, St. Louis, MO), and anti-Mannosidase II-Golgi marker was from ABCAM (Abcam Inc. MA).

### ATP assay

ATP level was measured using ATP Assay Kit. Human fetal neurons were incubated with Tat or rotenone, then the mitochondrial fraction was isolated by differential centrifugation. ATP was assayed using the ATP Determination Kit (Molecular Probes, Eugene, OR). This is a bioluminescence assay for quantitative determination of ATP using the recombinant firefly luciferase protein and substrate D-luciferin. Ten μL of the lysate were added to the ninety μL of the standard reaction solution, D-luciferin and 2.5 μL firefly luciferase (5 mg/mL). Bioluminescence was measured using a Luminometer (Femtomaster FB 12 luminometer, Zylux).

### ROS determination assay

Neuronal cells were cultured on the microscope cover glasses (Fisherbrand), then transfected or treated with Tat. After incubation with 1 mM MitoSOX red (Life Technologies) for 30 min, cells were analyzed by confocal microscopy. MitoSOX red signal was quantified using Image J.

### Superoxide dismutase (SOD) activity assay

SOD activity was measured in primary neuronal cells, using the OxiSelect SOD Activity Assay kit. Cytosolic fraction of neuronal cells was prepared and incubated with Xanthine Oxidase Solution for one hour at 37°C. Generated superoxide anions were measured at 490 nm. SOD activity was determined as the inhibition of chromogen reduction. Superoxide anion concentration is reduced in the presence of SOD, resulting in less colorimetric signal. Activity of SOD is shown in % of control.

### Statistical analysis

Data were analyzed using Excel software, Student’s t-test, and the Image J software (NIH). The error bars show the standard deviation from three independent readings from two experiments.

### Microscopy

Images of Tat-treated neuronal cells were captured using IPLAB software. Fluorescence staining for visualization of CFP-Tat protein and Tat deletion mutants was performed using blue (CFP) fluorescence. Human neurons were seeded in poly-L-Lysine coated glass slide chambers, and after 48 h incubation with 50 ng/mL of Tat protein, cells were analyzed by microscopy. Contrast and brightness were adjusted using Adobe Photoshop version 5.5. Original magnification for fluorescence images was 400x and for phase images was 200x.

## Results

### HIV-1 induced mitochondrial DNA damage in human cortical neurons and in PBMC

**Figure 1:**
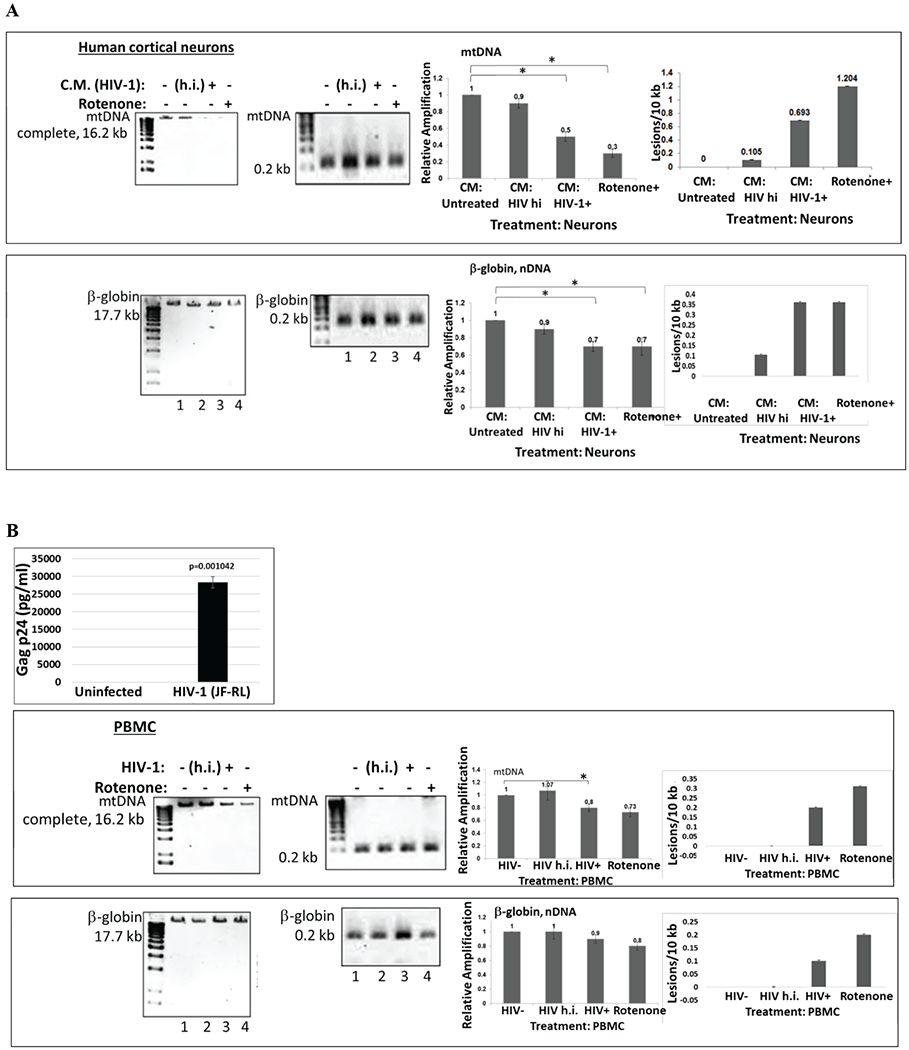
HIV-1 and HIV-Tat induced mitochondrial DNA damage in human cortical neurons and in PBMC. **A)** Human PBMC cells were first infected with HIV-1 or with heat inactivated HIV-1, for 5 days. Conditioned media from PBMC cells were used to treat neuronal cells for 24 hours, and total cellular DNA was isolated. QPCR was performed for both the mitochondrial DNA (complete mtDNA in 16.2 kb, or short mtDNA in 0.2 kb, used for the normalization; top panels) and β-globin nuclear DNA (nDNA) fragments (bottom panels). mtDNA and nuclear DNA damage was calculated and demonstrated as a Relative Amplification and as a Lesions/10 kb (right panels). Control cultures were incubated in media with rotenone, a drug that causes mtDNA damage. Representative images from agarose gel depicting the decrease in amplification of the 17.7-kb β-globin fragment (nuclear DNA or nDNA), (bottom panels) and the 16.2-kb mitochondrial fragment (top images). The decrease in relative amplification is shown by graphs. The data are expressed as the mean ± CD from a minimum of two biological experiments in which PCRs for mitochondrial (mtDNA) or nuclear DNA (nDNA) were performed in triplicates per experiment. Asterisks *indicate that the difference between treated or untreated samples is statistically significant (p<0.05). **B)** mtDNA damage in human PBMC cells infected with HIV-1 or heat inactivated HIV-1, for 5 days. The data are expressed as the mean ± SD (n=6) from two independent experiments in triplicates. p24 ELISA in PBMC cells infected with HIV-1 JR-FL strain to show efficient infection. ELISA was performed in triplicates and p-value was calculated using Student’s t-test. Asterisks *indicate that the difference between infected or uninfected samples is statistically significant (p<0.05).

We compared effects of HIV-1 infection of PBMC, or treatment of neurons with HIV-1 conditioned media on mtDNA damage. We demonstrate that both HIV-1 infection of PBMC (Figure 1B), or HIV conditioned media (Figure 1A) caused increased mtDNA damage in human cortical neurons and PBMC. We also demonstrate that although both mitochondrial and nuclear DNA were damaged, the mtDNA damage was stronger, compared to the effects of a positive control, rotenone treatment. Further, human PBMC cells were infected with either HIV-1 strain or with heat inactivated HIV-1 for 5 days. Conditioned media from PBMC were further used for the treatment of neuronal cells (right panels) for 24 hours, and genomic DNA (gDNA) was purified to perform mtDNA studies. qPCR was performed to generate both the mitochondrial (top bars) and β-globin fragments (bottom bars). Control cultures were incubated in media with rotenone. Representative images from agarose gel show the decrease in the level of amplified 17.7-kb β-globin fragment (*lower panels*) and the 16.2-kb mitochondrial fragment (*upper panels*). Short PCRs for mtDNA and nDNA were performed to normalize data obtained from the long PCRs. The decrease in relative amplification in HIV-1incubated or infected cells is shown in graphs. As a result, an increase in the lesions was demonstrated in the same samples on right panels. These findings clearly demonstrate that inactivated HIV-1 was unable to cause mtDNA damage. We also show that while HIV-1 cannot infect neurons, HIV-1 secreting molecules, including Tat and Vpr proteins, and also inflammatory cytokines and chemokines can play an important role in neuronal cell death.

### HIV-1 Tat and Vpr-induced mitochondrial DNA damage in human primary neuronal cells

**Figure 2:**
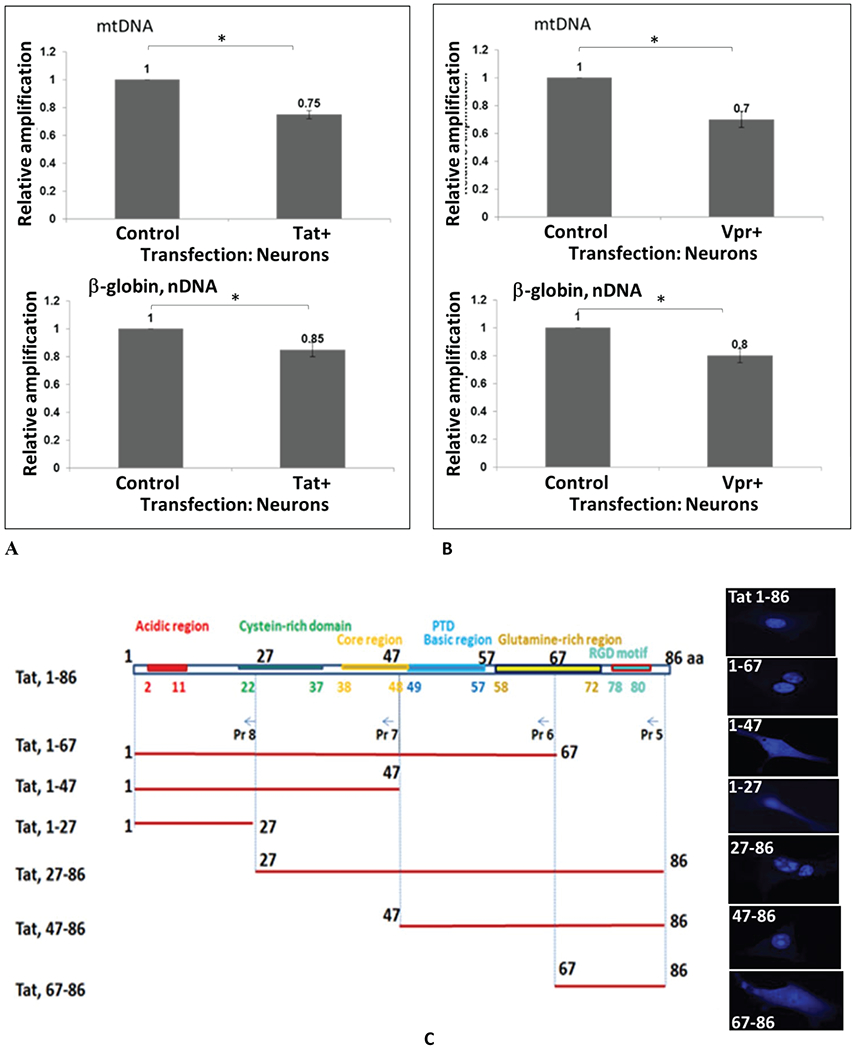

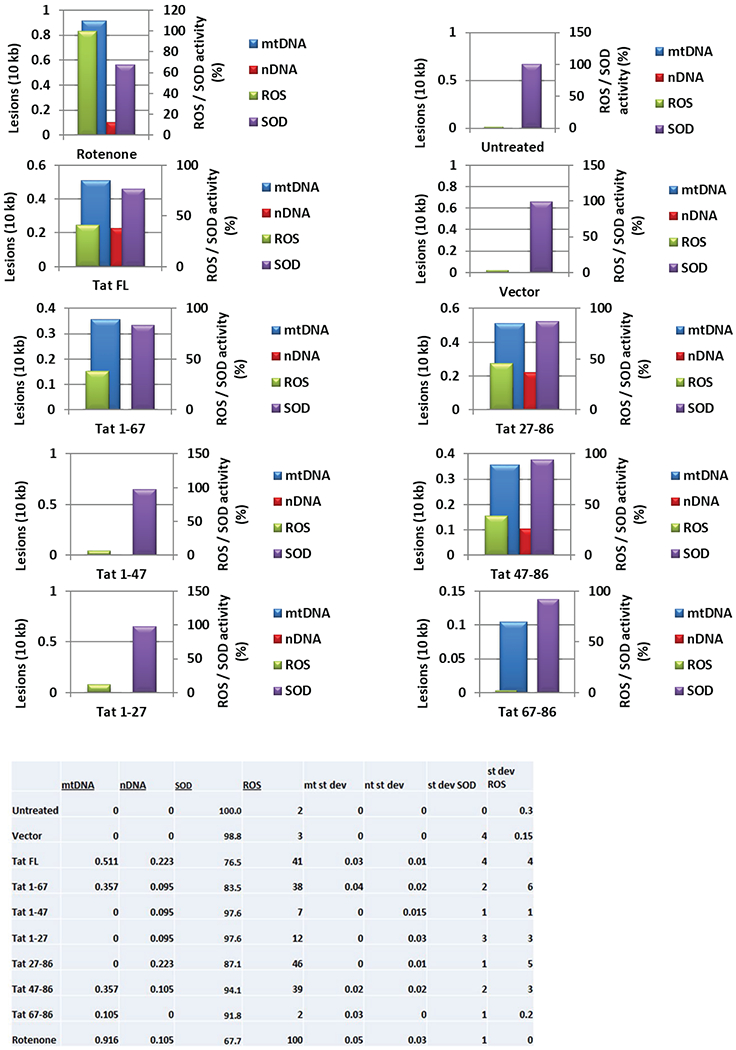
**A-C:** HIV-1 Tat and VPR-induced mitochondrial DNA damage in human primary neuronal cells. Cortical neurons were transfected with pECFP or pECFP-Tat **(A)** or VPR **(B)** for 48 hours. Total cellular DNA was isolated. QPCR was performed for both the mitochondrial (top bars) and nuclear β-globin fragments (bottom bars). The mtDNA and nuclear qPCR results (in fold change) are compared with control. The data represent the average of two independent experiments using triplicated samples). Asterisks *indicate that the difference between infected or uninfected samples is statistically significant (p<0.05). **C)** Tat deletion mutants were used in transfection of neuronal cells to identify Tat-responsive region in mtDNA damage induction. Graphical representation of Tat domains and deletion mutants (top panel). Cellular localization of each Tat- deletion mutant in transfected cells is shown on the right for each Tat variant. **D)** Tat deletion variants were studied in the mtDNA damage induction assays, and SOD and ROS activity. Table summarized data shown on the bottom.

Cortical neurons were transfected with pECFP or pECFP-Tat (Figure 2A) or Vpr (Figure 2B) for 48 hours. QPCR was performed for both the mitochondrial DNA (top panels) and nuclear β-globin fragments for nDNA (bottom panels). The mtDNA and nDNA qPCR results (relative amplification, in fold change) are compared to control. We demonstrate that both HIV-1 Tat and Vpr proteins cause mtDNA damage. To localize a responsive region within Tat, series of Tat deletion mutants were used in transfection reactions (Figure 2C). Graphical representation of Tat deletion mutants with or without a protein transduction domain (PTD) or a strong nuclear localization signal (NLS) containing 47-57 amino acid regions is shown on the left, and images of cells with a cellular appearance of each mutant are presented on the right. Our findings indicate that Tat deletion mutants without PTD are localized in the cytoplasm of transfected cells, while all peptides with PTD can be detected in the nucleus. mtDNA damage, nuclear DNA damage, SOD and ROS were assayed for each Tat deletion mutants, and results are shown in figure 2D. Table on the bottom summarizes data for all samples. High levels of mtDNA damage was detected in the samples treated with Rotenone, or transfected with full-length Tat, Tat (1-67), Tat (67-86), and Tat (47-86). Less nuclear DNA damage was observed in cells expressing the same Tat deletion mutants, while SOD activity was negatively correlated with mtDNA damage, and more mtDNA damage was found in cells, less SOD was detected.

Interestingly, not all mutants that caused an increase in mtDNA damage were also responsible for the inhibition of SO D, thus Tat mutant (47-86) had less effect on SOD activity, while caused mtDNA damage, suggesting that not only PTD, but also other regions within Tat may have an important role for SOD inactivation. As for ROS, full-length Tat and Tat (27-86) and Tat (47-86) had a strong elfect on both mtDNA damage and ROS, while Tat (27-86) surprisingly did not cause mtDNA damage, but negatively affected the accumulation of ROS. We suggest that full length Tat was important for the activation of mtDNA damage, ROS accumulation, and inhibition of SOD activity, while all other deletion mutants had partial effects in either events.

### Mitochondrial DNA damage in human primary neuronal cells, induced by recombinant HIV-1 Tat peptide or adenoviral Vpr

**Figure 3:**
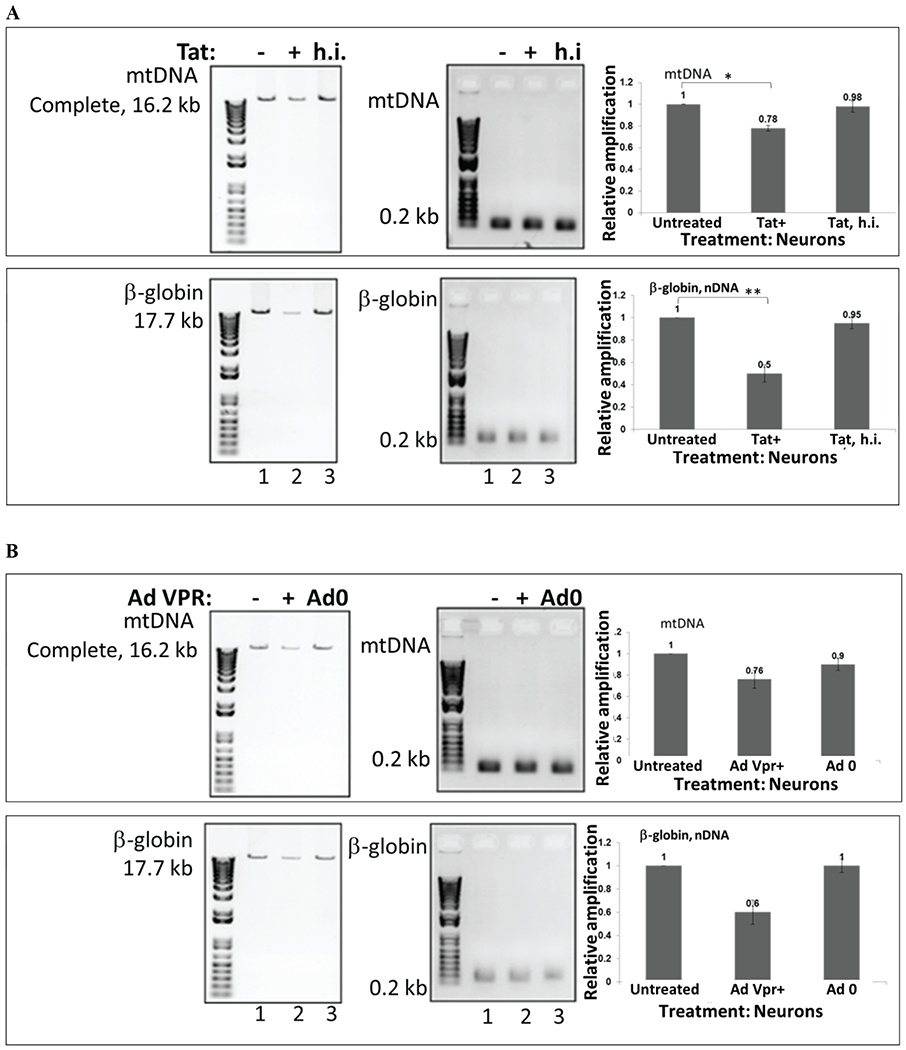
Mitochondrial DNA damage in human primary neuronal cells, induced by recombinant HIV-1 Tat peptide or adenoviral VPR. Cortical neurons were incubated with recombinant Tat or heat inactivated Tat **(A)** and adenoviral VPR **(B)** for 48 hours. Total cellular DNA was isolated, QPCR was performed for both the mitochondrial (top bars), and nuclear β-globin fragments (bottom bars). The mtDNA and nuclear qPCR results (in fold change) are compared with control. The data represent the average of two independent experiments (in triplicates for each sample). The analysis graph showed that compared with control, Tat induced nuclear DNA damage, while mitochondrial DNA damage was significantly lower, but both, mitochondrial and nuclear DNA damage were higher in cells incubated with VPR than controls (adeno-null treated cells).

Cortical neurons were incubated with recombinant full length Tat (101 amino acids) or heat inactivated Tat (Figure 3A) or adenoviral Vpr (Figure 3B) for 48 hours. Total genomic DNA was isolated and qPCR was performed for both the mtDNA (top bars) and nuclear β-globin fragments (bottom bars). The mtDNA and nuclear qPCR results are compared with control. The graph demonstrates that Tat treatment induced a strong nDNA damage, while less mtDNA damage was determined in comparison. Next, both mitochondrial and nuclear DNA damage was high in cells incubated with Vpr compared to controls (adeno-null treated cells). Our data suggest that either through transfection, or transduction, Tat causes mtDNA damage in neuronal cells. Previously, we confirmed that ultrapure Tat protein or short PTD-Tat (47-57 amino acids) was entering into neuronal cells or nucleus as early as after 40 minutes post-treatment [37,38]. Our and other research groups investigated features and characteristics of Tat protein to achieve an efficient transduction and to avoid degradation or cleavage prior its transportation into nucleus [39–43].

### Titration of doses of Tat and rotenone in incubation assays

**Figure 4:**
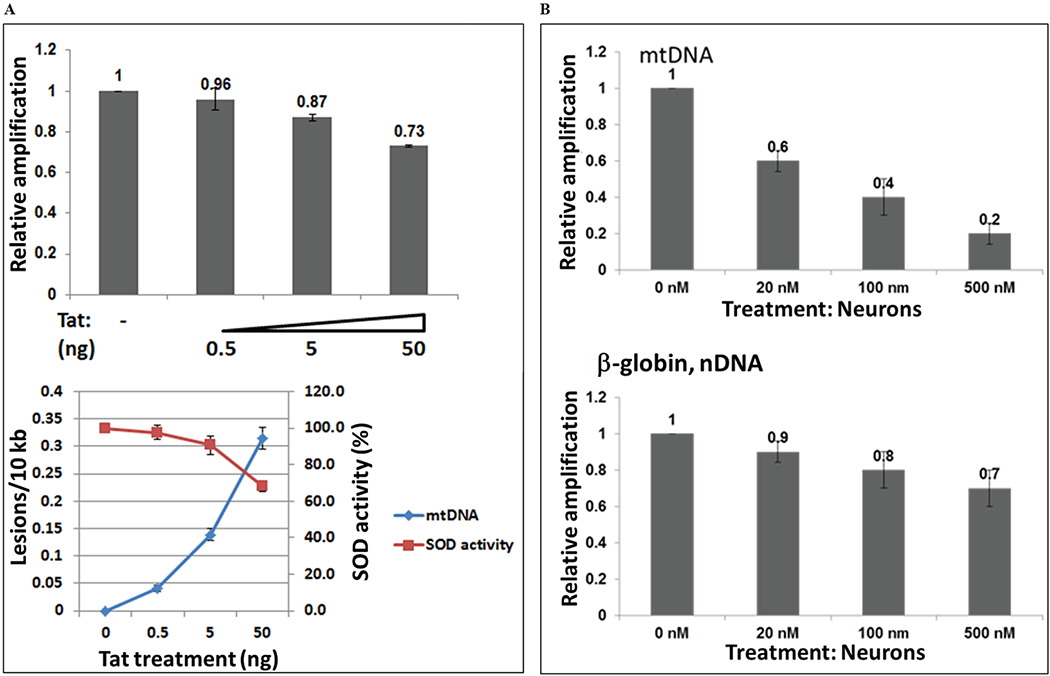
Titration of doses with Tat peptide or rotenone, and mtDNA damage and SOD activity. Mitochondrial DNA damage in human primary neuronal cells induced by increased concentrations of Tat 101 FL peptide from 0.5 to 50 ng, or 0.036 to 3.6 nM **(A)**, or by various concentrations of rotenone as specified (20to 500 nM), as a positive control **(B)**. Cortical neurons were incubated with various concentrations of Tat and rotenone, and a total cellular DNA was isolated and QPCR was performed for the mitochondrial and nuclear fragments. Graphs demonstrate mtDNA damage, SOD activity in panel A, and mtDNA damage and nuclear DNA damage in panel B.

Several concentrations of Tat (0.5 to 50 ng/mL) were used to treat neuronal cells to find out a lowest and an optimal concentration of Tat that can cause mtDNA damage or inhibition of SOD activity in human primary neuronal cells (Figure 4A). mtDNA damage was also induced by rotenone, as a positive control causing mtDNA damage (Figure 4B). Cortical neurons were incubated with various concentrations of rotenone (20 to 500 nM), as specified. Total genomic DNA was isolated and qPCR was performed for the mitochondrial and nuclear gene products. We found that although lower concentrations of Tat can also cause mtDNA damage, the optimal concentration of Tat that has a strong effect on mtDNA damage was 50 ng/mL, or 3.6 nM in incubation studies.

### Titration of incubation time with Tat and rotenone, and recovery assays

**Figure 5:**
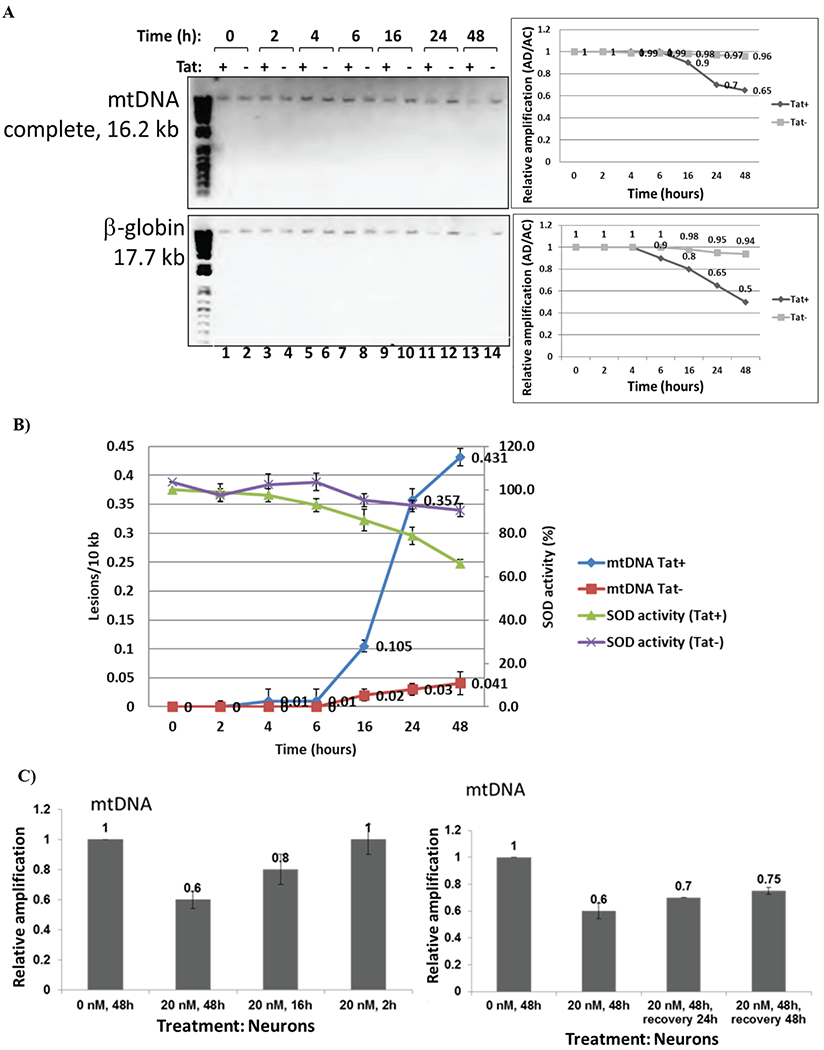
Titration of incubation time with Tat peptide or rotenone, and recovery assays. Mitochondrial DNA damage in human primary neuronal cells induced by Tat peptide at different times of incubation (A, B) or rotenone (C). **A)** Cortical neurons were incubated with Tat at different times of incubation (0 to 48 hours), as specified. Total cellular DNA was isolated and QPCR was performed for the mitochondrial and nuclear genes. Results for mtDNA and nDNA damage are shown in right panels. **B)** SOD activity in neurons for same time points (0 to 48 hours) of Tat treatment. **C)** mtDNA damage in neurons caused by Rotenone treatment of cells at different time points from 0 to 48 hours (top panels). Recovery assays from rotenone-caused mtDNA damage tested at 24 to 48 hours of rotenone removal (and replacement with a fresh media 24 h post-treatment) shown in bottom panels.

Fifty ng/mL (3.6 nM) of Tat were used to treat neuronal cells during various incubation times (0 to 48 hours), to find out an optimal time that Tat can cause mtDNA or nuclear DNA damage in human primary neuronal cells (Figure 5A), or inhibition of SOD activity (Figure 5B). Sixteen hours of Tat incubation was shown to be a time point with initial strong mtDNA damage detection. Six hours of incubation with Tat was a time pint to detect initial decrease in SOD activity. mtDNA damage was also induced by 20 nM of rotenone for 2, 16 or 48 hours of incubation (Figure 5C). Cortical neurons were incubated with rotenone (20 nM) at different incubation times, as specified. Total genomic DNA was isolated and qPCR was performed for the mitochondrial gene products (top graph). As 48 hours of rotenone incubation was an optimal time point when mtDNA damage was strongest (Figure 5C, top panel, bar 2), we next performed recovery assay, by removing the media with rotenone, and incubating cells in fresh media for another 24 and 48 hours. This assay will allow us to measure whether mtDNA damage that occurred in 48 hours after rotenone treatment, can be repaired, and if so, how soon or how long it will take for mitochondrial DNA to recover (Figure 5C, bottom). Data from the mtDNA damage measurements show that relative amplification of mtDNA increased from 0.6 to 0.7 after 24 hours of incubation in fresh media, indicating that mtDNA damage can still be repaired after 48 hours post-incubation with rotenone, and after 24 hours of replacing of the rotenone-containing media with fresh media. Furthermore, after next 24 hours (48 hours total) mtDNA damage was still repairing, as the relative amplification reached 0.75 compared to previous 0.6, or 0.7.

### HIV-1 Tat dysregulates mitochondrial energy metabolism in human neurons

**Figure 6:**
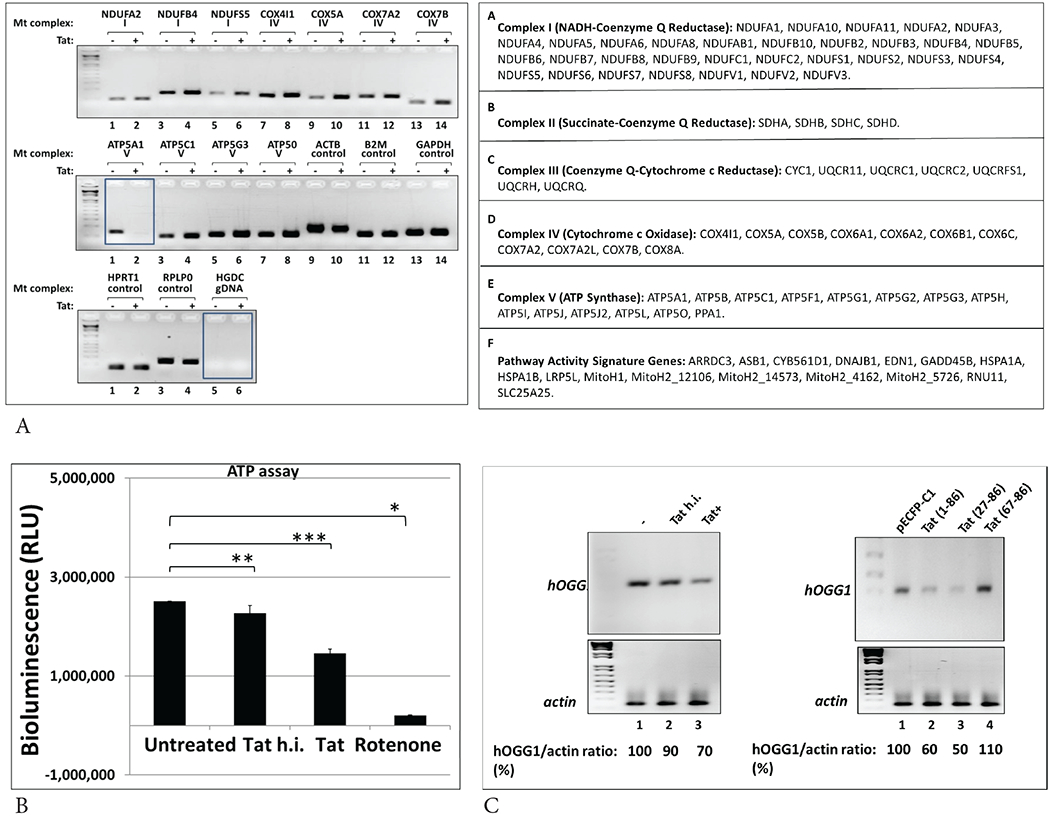
Effect of Tat on mitochondrial energy metabolism in human neurons. **A)** qRT-PCR assays were performed for genes involved in mitochondrial bioenergetics, using Mitochondria Energy Metabolism Arrays. Table on the bottom shows genes that represent all five Complexes (I to V). **B)** To confirm changes in ATP gene expression, ATP determination assay was performed in Tat treated neurons. Human primary neurons were treated with Tat, heat inactivated Tat (h.i.) or rotenone for 48 hours. The mitochondrial pellet was lysed in RIPA buffer, then the lysates were analyzed for ATP levels using ATP-luciferase assay. ATP levels were decreased significantly both in Tat or rotenone treated cells. Asterisks indicate that the difference between treated or untreated samples is statistically significant (p<0.05). Data represent the mean ± SD (n=6); *-p=0.05, **-p=0.01, ***-p=0.001. **C)** Treatment of neuronal cells with Tat (left panels) or transfection of cells with Tat expressing plasmid (right panels) causes inhibition of gene expression of mtDNA damage repair 8-oxoguanine DNA glycosylase-1 (OGG1), an enzyme that can repair 8-oxoG DNA lesion, studied by qRT-PCR. Our data demonstrate that Tat causes inhibition of OGG1 gene expression (70% or 60% a), which will interfere with the mtDNA damage repair mechanisms.

To investigate whether effects of Tat on mtDNA damage and SOD activity interferes with mitochondrial functions, we studied mitochondrial bioenergetics, using Mitochondria Energy Metabolism Arrays and qRT-PCR. This array includes 84 genes that represent all five mitochondrial complexes (I to V) (Figure 6A, right panels). Products of qRT-PCR were visualized by agarose gel electrophoresis, and images of gels are shown in right panels. While several genes representing all complexes were up- or down-regulated in Tat-treated cells, we were interested in particularly in ATP5A1 gene whose expression was strongly inhibited in the presence of Tat (Figure 6A, in box). To further confirm changes in ATP gene expression, we performed ATP assay in Tat treated neurons using ATP-luciferase assay (Figure 6B). ATP levels decreased significantly both in Tat or rotenone treated cells (bars 3 and 4). As an accumulation of mtDNA damage and ROS-induced mtDNA lesions correlates with decrease in *8-oxoguanine DNA glycosylase-1* (*OGG1*), an enzyme that can repair 8-oxoG DNA lesion, we also analyzed OGG1 gene expression (Figure 6C) in Tat-treated neuronal cells (left panels) or Tat-transfected cells (right panels), by qRT-PCR. Our data demonstrate that Tat causes inhibition of OGG1 gene expression (70% or 60% a), which will interfere with the mtDNA damage repair mechanisms.

### Tat increases the levels of apoptotic proteins in human neurons

**Figure 7:**
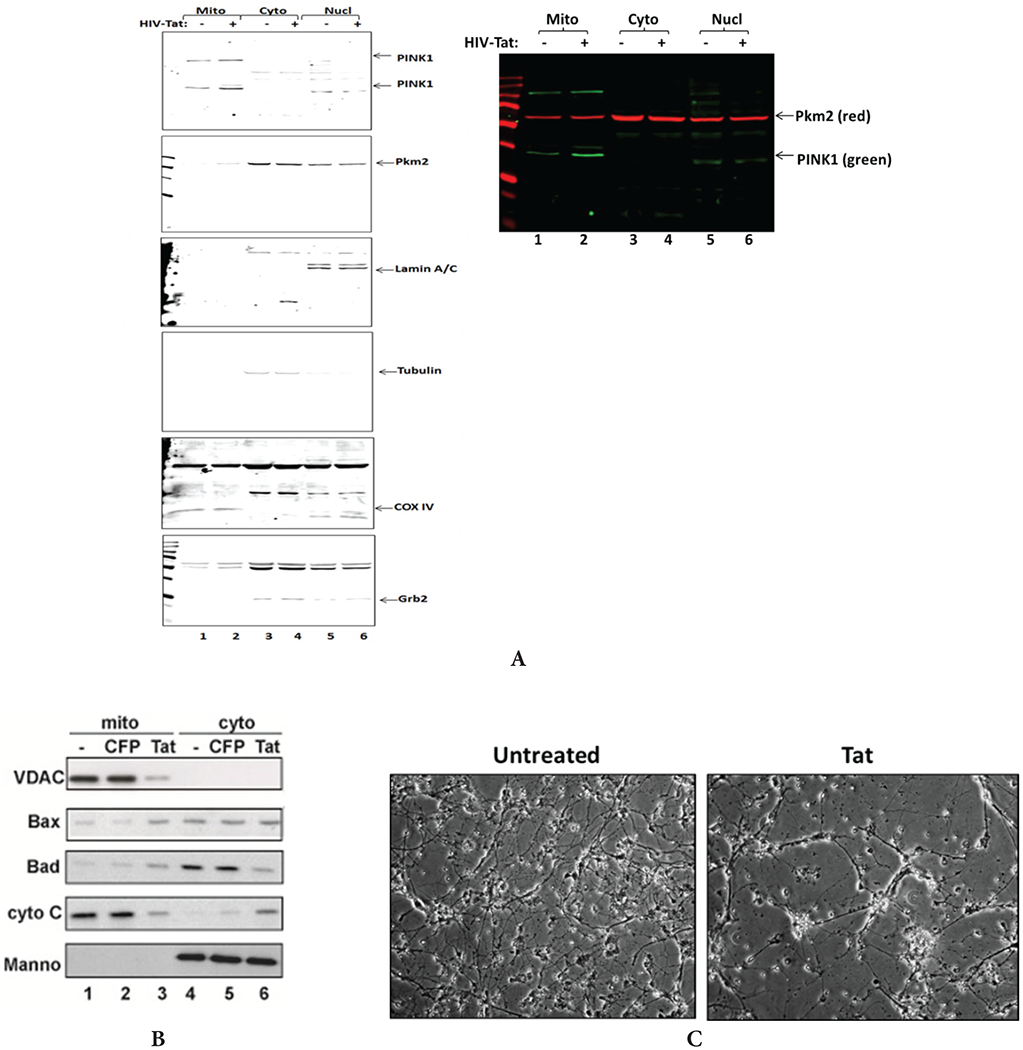
Tat increases the levels of apoptotic proteins and dysregulates mitochondrial proteins cellular localization in human neurons. **A)** Quantitative western blot assay was performed for PINK1 and PKM proteins in lysates from Tat treated neuronal cells. PINK1 and PKM are localizing at higher levels at the membranes of injured/damaged mitochondria. Cell fractionation was performed using mitochondrial isolation kit to separate mitochondrial, cytoplasmic and nuclear fractions. COX IV was used as a mitochondrial fraction control; Grb2 and Tubulin were used as cytoplasmic fraction purity and a loading control, and Lamin A-as a nuclear fraction control. **B)** Tat expression in transfected neurons activates apoptotic Bax and Bad and inhibits outer membrane VDAC in mitochondrial fraction, and leads to the translocation of apoptotic Cytochrome C from mitochondria to cytoplasm. **C)** Images of neuronal cells with or without Tat.

To study whether Tat-caused induced mtDNA damage and mitochondrial bioenergetics affect apoptotic events in mitochondria of neuronal cells, we performed q-western blot assay for apoptotic proteins, PTEN-induced kinase 1 (PINK1) and Pyruvate Kinase M2 (PKM2) proteins in lysates from Tat treated neuronal cells (Figure 7). Cell fractionation for mitochondrial, cytoplasmic and nuclear fractions demonstrated an increased level of PINK1 in mitochondrial fraction in Tat-treated neuronal cells (Figure 7A, lane 2), suggesting an increase in mitochondrial injury in the presence of Tat in neuronal cells. Several apoptotic proteins including Bax and Bad also were shown to be increased in mitochondrial fraction (Figure 7B). In contrary, other two proteins, an outer membrane VDAC and Cytochrome C were decreased in mitochondrial fraction (Figure 7B, lane 3), while there was an noticeable increase in Cytochrome C in the cytoplasmic fraction of Tat-treated cells (Figure 7B, panel 4, lane 6). In addition to mitochondrial DNA damage and activation of apoptotic proteins, Tat treatment caused neuronal cell injury and cell loss (Figure 7C).

Our data suggest a strong involvement of Tat in mitochondrial DNA damage, changes in mitochondrial energy metabolism and neuronal apoptosis. This is the first observation for HIV-Tat induced mitochondrial DNA damage in human neurons.

## Discussion and Conclusion

In this work, we showed an effect of HIV-1 and HIV-1 proteins on mtDNA damage in primary human neuronal cultures that was seen in both cortical neurons and PBMC. Our neuronal system presents an ideal model to study mitochondrial DNA damage affected by HIV-1 or HIV-1 proteins with the potential of further translational studies. Firstly, we demonstrated that the use of conditioned media collected from HIV-infected PBMC cells could cause mtDNA damage in human neurons. Conditioned media contains HIV-1 proteins and other active molecules, including pro-inflammatory cytokines and chemokines that can have toxic effects on neuronal cells. Thus, these secreting proteins, including Tat and Vpr can be transduced into neuronal cells and have an influence on mtDNA damage and neuronal apoptosis.

To date there has not been much knowledge about neuronal mtDNA during HIV-1 infection. Several studies showed that Tat affects on itochondrial bioenergetics, although mitochondrial DNA damage has not been demonstrated in human neurons. The effect of HIV-1 or Tat on mtDNA levels and mitochondrial function in PBMC was recently described [22,44]. Effects of Tat on mitochondrial bioenergetics were demonstrated in cardiomyocytes [23]. Furthermore, the effects of Tat on mitochondrial genes and DNA were shown in Jurkat cells [22]. Other studies discuss the effects of Tat on DNA damage in human peripheral blood B-lymphocytes *via* mitochondrial ROS production [10]. Finally, it was shown that Tat induces mitochondrial membrane permeabilization in human liver and heart [24]. Thus, no direct effects of Tat or HIV-1 infection on mtDNA damage were demonstrated in human neurons. Our goal here was to examine the effects of Tat on neuronal mitochondrial DNA damage in human neurons, as one of potential events in neurodegeneration and neurological complications in NeuroHIV and AIDS. Studies of apoptotic and mitochondrial proteins, including PINK1 and PKM2, is also important to confirm mitochondrial damage. PINK1, a mitochondrial kinase that protects cells from stress-induced mitochondrial dysfunction, is known to be translocated to the damaged mitochondria in the complex with Parkin, and accumulated at higher levels at the membranes of mitochondria during injury [4,11,45] while mitochondrial PKM2 can regulate oxidative stress-induced apoptosis [46]. Further studies were necessary to investigate neuronal mtDNA damage during infection, to uncover the role of HIV and HIV-1 proteins, Tat or Vpr on mtDNA damage and mitochondrial bioenergetics. To answer this question, we used human primary neuronal culture model and human PBMC to study mtDNA damage during HIV-1 infection, or caused by HIV Tat and Vpr proteins.

Thus, we demonstrate novel findings on mitochondrial DNA damage in human neurons. In our future studies, it will be also important to investigate whether Tat can directly damage mtDNA *in vitro*, while other mitochondrial proteins will not interfere with HIV-Tat protein activity. These studies collectively pave the road for the design of therapeutic modalities for the treatment of neurological deficits inflicted by HIV-1 infection.
